# Anti-HCV Tannins From Plants Traditionally Used in West Africa and Extracted With Green Solvents

**DOI:** 10.3389/fphar.2021.789688

**Published:** 2022-01-28

**Authors:** Moussa Bamba, Simon Bordage, Marie-Emmanuelle Sahuc, Sophie Moureu, Jennifer Samaillie, Vincent Roumy, Peggy Vauchel, Krasimir Dimitrov, Yves Rouillé, Jean Dubuisson, Fézan Honora Tra Bi, Karin Séron, Sevser Sahpaz

**Affiliations:** ^1^ UFR Sciences de La Nature, Université Nangui Abrogoua, Abidjan, Côte d'Ivoire; ^2^ Université de Lille, Université de Liège, Université de Picardie Jules Verne, JUNIA, UMRT 1158 BioEcoAgro, Métabolites Spécialisés D’origine Végétale, Lille, France; ^3^ Univ Lille, CNRS, INSERM, CHU Lille, Institut Pasteur de Lille, U1019-UMR 9017-CIIL-Center for Infection and Immunity of Lille, Lille, France

**Keywords:** antiviral, tannins, traditional medicine, hepatitis C, Côte d'Ivoire, sustainable extractions

## Abstract

Millions of people are still infected with hepatitis C virus (HCV) nowadays. Although recent antivirals targeting HCV proteins are very efficient, they are not affordable for many people infected with this virus. Therefore, new and more accessible treatments are needed. Several Ivorian medicinal plants are traditionally used to treat “yellow malaria”, a nosological category including illness characterized by symptomatic jaundice such as hepatitis. Therefore, some of these plants might be active against HCV. An ethnobotanical survey in Côte d’Ivoire allowed us to select such medicinal plants. Those were first extracted with methanol and tested for their anti-HCV activity. The most active ones were further studied to specify their IC_50_ and to evaluate their toxicity *in vitro*. Greener solvents were tested to obtain extracts with similar activities. Following a phytochemical screening, tannins of the most active plants were removed before re-testing on HCV. Some of these tannins were identified by UPLC-MS and pure molecules were tested against HCV. Out of the fifteen Ivorian medicinal plants selected for their putative antiviral activities, *Carapa procera* DC. and *Pericopsis laxiflora* (Benth. ex Baker) Meeuwen were the most active against HCV (IC_50_: 0.71 and 0.23 μg/ml respectively) and not toxic for hepatic cells. Their crude extracts were rich in polyphenols, including tannins such as procyanidins A2 which is active against HCV. The same extracts without tannin lost their anti-HCV activity. Replacing methanol by hydro-ethanolic solvent led to tannins-rich extracts with similar antiviral activities, and higher than that of aqueous extracts.

## Introduction

Hepatitis C is a major cause of chronic hepatitis often associated with complications, such as liver cirrhosis and hepatocellular carcinoma (Levrero, 2006; Messina et al., 2015). It is estimated that approximately 71.1 million people are infected with hepatitis C virus (HCV) worldwide (Polaris Observatory HCV Collaborators, 2017). The prevalence of HCV infection varies considerably between countries. The adult HCV seroprevalence was estimated at 2.2% in Côte d’Ivoire and can be greater than 10% in some countries like Egypt (Riou et al., 2016). To date, no vaccine is available (Tarr et al., 2015). In the last decade, treatments with direct-acting antiviral (DAA) agents targeting viral proteins have been established. These therapies with high rates of sustained viral response against all HCV genotypes and very few side effects are driven by DAAs that target viral proteins NS3/4A protease, NS5A, and NS5B polymerase (Falade-Nwulia et al., 2017). However, there is a risk of selecting viral variants resistant to DAAs leading to failure of the therapy, which will require new treatments (Pawlotsky, 2016). Moreover, these treatments are very expensive and will benefit only a very small proportion of patients (Graham and Swan, 2015). The use of plant extracts or compounds isolated from plant extracts should render the therapy more accessible to many patients in developing countries. Therefore, the search for such compounds and extracts is still needed to treat the vast majority of patients in the coming decades.

Natural products are largely used for healthcare worldwide, and remain an important source of inspiration for medicinal chemistry nowadays (Newman and Cragg, 2020). Numerous DAAs from natural origins are now described and are able to target different steps of the HCV infectious cycle. Many plant extracts and natural products with such antiviral activities have been reviewed these last years (Patil et al., 2016; Jardim et al., 2018; Seron, 2018). In this context, crude extracts from plants used in traditional medicine are promising sources of antiviral molecules.

In Côte d’Ivoire, several medicinal plants are traditionally used to treat “yellow malaria”, a nosological category including illness characterized by symptomatic jaundice such as hepatitis. Based on an ethnobotanical survey and a bibliographic search, 15 plants were selected in Côte d’Ivoire. They were screened *in vitro* for their activity on HCV and the two most active ones were selected and further studied. For this antiviral screening, methanol was first used to obtain crude extracts for each plant. However, this toxic solvent can obviously not be used in traditional medicine. In addition, a sustainable approach was carried out. Therefore, methanol was replaced by hydro-ethanolic solvents and leaves were used rather than bark or roots. The composition of obtained extracts was investigated in order to elucidate which types of components might contribute to the anti-HCV activity.

## Material and Methods

### Chemicals, Culture Medium and Antibodies

Solvents used for extractions were ethanol and methanol (>99% purity; VWR France), and ultrapure water prepared with an Integral 5 Milli-Q system (Merck Millipore). Ethyl acetate (Fisher), acetic acid and formic acid (Carlo Erba) were used for TLC.

Dulbecco’s modified Eagle’s medium (DMEM), Opti-MEM, phosphate buffered saline (PBS), glutamax-I, and fetal bovine serum were purchased from Invitrogen (Carlsbad, CA). 4′,6-Diamidino-2-phenylindole (DAPI) was from Molecular Probes (Thermo Fischer Scientific, Waltham, United States).

Delphinidin chloride was from Extrasynthèse (Lyon, France) and was >96% pure. Boceprevir was kindly provided by Philippe Halfon (Hôpital Européen, Laboratoire Alphabio, Marseille, France). Procyanidins A1, A2, B1, B2 and B3 were >96% pure and from Phytolab (Germany). All these compounds were tested on Huh-7 cells. In addition, several other compounds were used as standards for TLC or UPLC: quercetin and quinin were from Merck (Germany), gallic acid, stigmasterol and glycyrrhetic acid were from Extrasynthése (France), (−)-epicatechin, (+)-catechin, aloin and ellagic acid were from Sigma (Germany), (−)-EGC was from Chromadex (United States), (−)-EGCG was from Selleckchem (United States).

The anti-E1 monoclonal antibody (A4) (Dubuisson et al., 1994) was produced *in vitro* by using MiniPerm apparatus (Heraeus, Hanau, Germany). Cy3-conjugated goat anti-mouse IgG was from Jackson Immunoresearch (West Grove, PA, United States).

### Ethnobotanical Survey

The ethnobotanical survey was conducted in the Bafing region, which is situated in North-West Côte d’Ivoire and corresponds to the Touba department. It covers a total land area of 8,650 km^2^ and shares boundaries with the Odienné department in the North and the Séguéla and Biankouma departments in the East and the South respectively. In the West, it shares boundaries with the Republic of Guinea. It is one of the most distant Ivorian regions from the capital city Abidjan. Therefore, people are still more dependent on traditional medicine compared to people living closer to cities. In this region, traditional healers could be assigned in at least one of these 3 groups: 1) “Bouêti”: they are plain healers, who know medicinal plants and treat common illnesses; they are the first medical resort in this region; 2) “Kamon” or marabout: they heal with natural products according to the Coran; 3) “Komanti”: they heal with natural products and talismans. Traditional healers could also belong to two of these groups: “Kamon-Bouêti” and “Komanti-Bouêti”. Seven of them (5 “Bouêti”, 1 “Komanti-Bouêti” and 1 “Kamon-Bouêti”) were met three times during one month of non-structured interviews, in the city of Touba, the Mahanan village and the Ouintoulo camp. Religion in Côte d’Ivoire is diverse and the main religions are Islam, Christianity (primarily Roman Catholic) and various indigenous religions. The Survey considered the use of plants against a group of diseases called “yellow malaria”, which is a rough translation for diseases that cause icterus. This nosological category include malaria but also other illness characterized by symptomatic jaundice as hepatitis, obstruction of the bile duct, hemolysis. Traditional healers were asked about the illness they cured with plants, the name of these plants, the part used, how they prepared them and the mode of application.

### Plant Material and Extractions

The 15 plants were collected in the wild during the morning. They were then cleaned and air-dried at constant temperature (26°C) for 1–2 weeks at the Nangui Abrogoua University (Abidjan). They were authenticated by a botanist at the Centre National de Floristique (CNF), University of Félix Houphouët Boigny de Cocody (Abidjan), by comparing their morphological characters to a flora. Voucher specimens were deposited in an Herbarium at the CNF. The voucher numbers and the traditional uses of the investigated species are presented in Table 1. Their names have been checked with “The World Flora Online” (http://www.worldfloraonline.org/- accessed October, 2021). Each plant was then powdered and stored in the dark until extractions. For each plant, 20 g of dried powder were mixed with 100 ml methanol for 24 h. After filtration, the grounds were extracted again twice in the same way. The 3 resulting filtrates were combined and dried under vacuum at 40°C.

**TABLE 1:** Ivorian plants traditionally used by the surveyed healers to treat yellow malaria.

Scientific name (Plant family)	Mahouka name (and English one), voucher numbers	Traditional healer uses	Plant part used and drug preparation	References for related uses
*Alstonia boonei* De Wild. (Apocynaceae)	Kouôgbêfa (Cheese wood), UCJ001553	White malaria, yellow malaria, fever	Leaf decoction (tea and bath)	Odugbemi et al. (2006), Tsabang et al. (2012), Ishola et al. (2014), Malan et al. (2015)
*Anogeissus leiocarpa* (DC.) Guill. and Perr. (Combretaceae)	Kéhété (African birch), UCJ002880	Yellow malaria	Bark decoction (tea and bath)	Adamu et al. (2005), Diarra et al. (2015)
*Carapa procera* DC*.* (Meliaceae)	Goué (African crabwood), UCJ012269	Stomach ache, yellow malaria	Leaf decoction (tea and bath)	Jiofack et al. (2009)
*Combretum collinum* Fresen*.* (Combretaceae)	Gnangbaha (Weeping bushwillow), UCJ002910	Generalised faintness, infected wound, yellow malaria	Leaf decoction (tea, bath and skin application); crushed leave (skin application)	Tshikalange et al. (2016)
*Mallotus oppositifolius* (Geiseler) Müll. Arg. (Euphorbiaceae)	Sêgbô, UCJ006172	Buruli ulcer, white malaria, yellow malaria, female infertility, diarrhoea, urinary tract infection	Leaf and bark decoction (tea and bath); Crushed leave (skin application)	Bremer Christensen et al. (2015)
*Momordica charantia* L*. *(Cucurbitaceae)	Foho faha (Bitter melon), UCJ004434	White malaria, yellow malaria, labour trouble, abortion, jaundice	Leaf decoction (bath and cleansing)	Kamaraj et al. (2012)
*Paullinia pinnata* L*.* (Sapindaceae)	Miannonmon (Tietie), UCJ016410	Erectile dysfunction, yellow malaria	Leaf decoction (tea): Vine (tooth cleaning)	Okpekon et al. (2004)
*Pericopsis laxiflora* (Benth. ex Baker) Meeuwen (Fabaceae)	Kooko (False dalbergia), UCJ009931	Fever, stiffness, yellow malaria	Branch and bark decoction (bath)	Asase et al. (2005), Bla et al. (2015)
*Periploca nigrescens* Afzel. (Apocynaceae)	Nonfôni (Afrian parquetina), UCJ014293	White malaria, yellow malaria, chronic wound, abortion	Leaf decoction (tea and bath); Crushed leave (skin application and cleansing)	Neuwinger, (1996)
*Pseudarthria hookeri* Wight and Arn. (Fabaceae)	Sougba’vin (Pink velvet bean), UCJ010930	Fever, yellow malaria	Leaf decoction (tea and bath)	Stangeland et al. (2011)
*Rauvolfia vomitoria* Afzel*.* (Apocynaceae)	Kouôyôli (Poison devil’s-pepper), UCJ002178	White malaria, yellow malaria, fever	Leaf decoction (tea and bath)	Rusaati, (2021)
*Sarcocephalus latifolius* (Sm.) E.A.Bruce (Rubiaceae)	Bêtou (African peach), UCJ015407	Fever, stiffness, yellow malaria	Leaf decoction (tea and steam bath)	Diarra et al. (2015), Agbodeka et al. (2016), Haidara et al. (2016)
*Terminalia macroptera* Guill. and Perr. (Combretaceae)	Kouhoman, UCJ003170	White malaria, yellow malaria	Branch and bark decoction (tea and bath)	Pham et al. (2011), Traore et al. (2013)
*Trichilia monadelpha* (Thonn.) J.J.de Wilde (Meliaceae)	Souafinza, UCJ012340	Stomach ache, infertility, yellow malaria	Leaf and bark decoction (tea, bath and cleansing)	Oppong Bekoe et al. (2020)
*Uapaca togoensis* Pax (Phyllanthaceae)	Somon’goh, UCJ006467	General fatigue, stiffness, yellow malaria	Leaf decoction (tea and bath), with *Terminalia glauscesens* and *Pseudarthria hookeri*	Koné et al. (2004)

*C. procera* and *P. laxiflora* were also extracted with water-ethanol and water-methanol mixtures (0–100%) as solvent. For each plant, extractions were carried out in 100 ml flasks with 2 g of dried powder and 40 ml of solvent. Flasks were placed for 2 h in a shaking incubator (Multitron II Infors HT) in order to maintain agitation at 160 rpm and temperature at 20°C. These extractions were carried out in triplicates. Final extracts were then centrifuged for 10 min at 10,000 tr. min^−1^ and 4°C and the supernatants were stored at −18°C in the dark until use. 10 ml of supernatant were used to determine dry mass yields (measurements made in duplicate, each one with 5 ml of supernatant) with an infrared moisture analyzer (Precisa XM60). The remaining volume of supernatant was freeze-dried (freezedryer Heto PowerDry PL9000 Thermo Fisher Scientific) so as to get dehydrated extract for further analysis. Evaporation under vacuum was carried out prior to freeze-drying in the case of supernatants containing ethanol or methanol (rotary evaporator Heidolph instrument, GmbH and Co. KG).

All the dried crude extracts or isolated compounds were resuspended in DMSO before antiviral assays. Plant extracts were dissolved in DMSO to reach a final concentration of 25 mg/ml. Delphinidin and boceprevir were also resuspended in DMSO at a final concentration of 100 mM. Serial dilutions in DMEM cell culture medium were then performed to reach the final concentration mentioned for each experiment.

### Phytochemical Screening

Phytochemical screening was performed with thin layer chromatography using pre-coated silica gel plates (Alugram Xtra Sil G/UV254, Machery-Nagel). The solvent system was EtOAc/Acetic acid/Formic acid/H_2_O (100:11:11:20 v/v). For each extract, 10 µL (5 mg/ml) were spotted on the TLC plate. The same revealers as in Santos *et al.* (Santos et al., 2018) were used, except for total and condensed tannins, for which ferric chloride (Oliveira et al., 2015) and DMACA (Lea, 1978) (Sigma-Aldrich) were used. Folin-Ciocalteu reagent (VWR chemicals) was also used to reveal all the polyphenols. The following standards were used as controls on TLC plates: quercetin, (+)-catechin, gallic acid, aloin, stigmasterol, glycyrrhetic acid and quinin.

### Tannin Removal

To quantify tannins and other phenolics in our extracts, Wiesneth and Jürgenliemk, (2017) that is based on the Folin-Ciocalteu method of the European Pharmacopeia (2.8.14) was adapted. Briefly, 1.5 ml of water was first added to 5 mg of each extract to dissolve them in water bath (95°C) during 30 min. These were centrifuged at 2,500 rpm for 5 min, and the supernatants were the “total phenolic solutions” (TPS). For each TPS, 10 mg hide powder was added to an aliquot of 0.5 ml. These preparations were stirred and further incubated without light for 60 min while shaking. The supernatants after centrifugation (2,500 rpm, 5 min) resulted in the solutions containing phenols without tannins (TPS-T). The removal of tannins was checked with TLC revealed by the DMACA reagent by comparing TPS (blue spots) and TPS-T (no spot).

### Polyphenols and Tannins Quantification

For total phenolics and tannins quantification, Wiesneth and Jürgenliemk microplate method was used. Briefly, except for the outer wells filled with water, 20 µL of sample (TPS, TPS-T), or gallic acid (Extrasynthese, France) for calibration curve were added in each well. Then 100 µL of a 1/10th dilution of Folin-Ciocalteu’s reagent was added and shaken without light for four minutes. Finally, 80 µL of a 10.6%-solution of Na_2_CO_3_ anhydrous in water was added to the samples. Measurement of absorbance at 690 nm at room temperature was achieved after 90 min using a Spectrostar Nano (BMG Labtech) spectrophotometer.

### Extracts Characterization by UPLC-UV-MS

Ultra-High Performance Liquid Chromatography (UPLC) analyses were performed on an Acquity UPLC H-Class system (Waters, Guyancourt, France) coupled with a Diode Array Detector (DAD) and a QDa ESI-Quadrupole Mass Spectrometer. All data were acquired and processed using Empower 3 software. Separation was achieved using an ACQUITY UPLC^®^ BEH phenyl 1.7 µm (2.1 × 100 mm) column (Waters, Milford MA). Gradient elution was performed with (A) 0.1% formic acid in water and (B) 0.1% formic acid in acetonitrile at a flowrate of 0.3 ml/min. The amount of B was increased from 5% for the initial 1 min to 30% at 9 min, and to 100% at 9.5 min. The condition was then kept at 100% B until 10.5 min. Finally, the column was re-equilibrated to 5% B in 0.5 min and maintained for an additional 2 min, resulting in a total run time of 13 min. Column oven and auto-sampler were set at 30°C and 15°C respectively, and 4 µL of each sample (1 mg/ml in MeOH/water) were injected. Analytes were monitored using UV detection (l range: 190–790 nm, resolution: 1.2 nm) and MS-Scan from 100 to 1,000 Da (both in positive and negative mode). Cone voltage was set at 15 V. Probe temperature was 600°C. Capillary voltage was 0.8 kV. The main tannins and anthocyanins were identified based on the retention time of the commercial standards and their mass spectra (Supplementary Table S1).

### Antiviral Assays

#### Cells and Culture Conditions

Huh-7 cells, established from a human hepatocellular carcinoma (Nakabayashi et al., 1982), were grown in DMEM supplemented with glutaMAX-I and 10% fetal bovine serum (complete culture medium) in an incubator at 37°C with 5% CO_2_.

#### Virus and Infectivity Assays

A modified JFH1 strain (Japanese Fulminant Hepatitis-1, genotype 2a) containing cell culture adaptive mutations (Delgrange et al., 2007; Goueslain et al., 2010) was used. JFH1 was kindly provided by T Wakita (National Institute of Infectious Diseases, Tokyo, Japan). The viral stocks were produced in Huh-7 cells. Huh-7 cells were infected with a pre-stock of HCVcc in flasks. After 24, 48 and 72 h the supernatants of flasks were collected. The titer of the stock was 5 × 10^6^ focus forming unit (ffu)/mL for JFH1. For infection assay, Huh-7 cells, 6,000/well, seeded in 96-well plate were inoculated with HCVcc at a multiplicity of infection (MOI) of 0.8 during 2 h at 37°C then the inoculum was removed and cells were incubated in complete culture medium for 28 h at 37°C. Compounds were added to cells for 2 h at 37°C in the presence of the virus (inoculation), and for 28 h at 37°C after virus removal (post-inoculation). Cells were fixed with ice-cold methanol and subjected to immunofluorescent detection of viral E1 envelope protein.

#### Immunofluorescent Detection Assay

HCVcc infected cells grown in 96-well plates were processed for immunofluorescence detection of viral proteins, as previously described (Rouillé et al., 2006). Nuclei were stained with 1 μg/ml of DAPI, and infected cells were detected by immunofluorescent labeling of E1 envelope glycoprotein (HCV), followed by Cy3-conjugated anti-mouse secondary antibody. For quantification of infection, confocal images were recorded with an automated confocal microscope IN Cell Analyzer 6,000 (GE Healthcare Life Sciences) using a ×20 objective with exposure parameters 405/450 nm and 561/610 nm. Six fields per well were recorded. Each image was then processed using the Colombus image analysis software (Perkin Elmer). Nuclei were first segmented and the cytoplasm region was extrapolated based on the DAPI staining. Objects with a specific and predefined size were defined as cells. The ratio of infected cells over total cells represents the infection rate. For each virus, the number of cells per well and MOI were determined in order to have 30–40% of infected cells in control experiments with no inhibitor. Infection rates in DMSO controls were expressed as 100%.

#### Cytotoxicity Assay

Huh-7 cells were plated in 96-well plates at a density of 6,000 cells/well and then were incubated the next day in 100 μL of culture medium containing increasing concentrations of plant crude extracts for either 24, 48, or 72 h. An MTS [3-(4,5-dimethylthiazol-2-yl)-5-(3-carboxymethoxyphenyl)-2-(4-sulfophenyl)-2H-tetrazolium]-based viability assay (Cell Titer 96 Aqueous non-radioactive cell proliferation assay, Promega) was performed as recommended by the manufacturer. The absorbance of formazan at 490 nm is detected using an ELISA plate reader (ELX 808 Bio-Tek Instruments Inc.). Each measure was performed in triplicate.

## Results and Discussion

### Antiviral Plant Selection

During our ethnobotanical survey, 15 plants were identified as commonly used to treat “yellow malaria” and were thus selected (Table 1). Indeed, this symptom could correspond to icterus, which could be caused by hepatitis. Since HCV is quite common in Côte d’Ivoire, and can cause icterus in some cases, some of these 15 plants might have anti-HCV activity. However, not to mention other important considerations such as cultural aspects in traditional medicine, jaundice can be caused by other hepatitis (either viral or not) or many other diseases. For instance, malaria, which is widespread in Côte d’Ivoire, can cause hemolysis which in turn can lead to icterus. Other symptoms such as anemia can be observed and described in “white malaria” (Traore et al., 2021). In fact, many of the plants screened here for putative antiviral activity have been reported to be used against malaria and/or have shown antiplasmodial activity (Table 1). For instance, leave or bark decoction of *Alstonia boonei* De Wild. are used to treat malaria by Ehotile people in Côte d’Ivoire (Malan et al., 2015) and also in Nigeria (Ishola et al., 2014) and Cameroon (Tsabang et al., 2012). *Anogeisus leiocarpa* (DC.) Guill. and Perr. was quoted as one of the most used plant to treat malaria in an ethnobotanical survey in Mali (Diarra et al., 2015). *Carapa procera* DC. extracts had antiplasmodial activity *in vitro* (Sarpong et al., 2016), and in a more recent study, they had anti-malaria activity on mice (Koama, 2021). Other plant parts were also reported to be used against malaria or have shown antiplasmodial activity: *Combretum collinum* Fresen*, Momordica charantia* L.*, Paulinia pinnata* L.*, Pericopsis laxiflora* (Benth. ex Baker) Meeuwen*, Rauvolfia vomitoria* Afzel.*, Sarcocephalus latifolius* (Sm.) E.A.Bruce and *Terminalia macroptera* Guill. and Perr. (Table 1). In our sustainable approach, only plant leaves were used for further studies, although other parts of the plants such as bark were traditionally used in some cases. For instance, the stem bark of *Pericopsis laxiflora* (Benth. ex Baker) Meeuwen was already reported to be traditionally used in Côte d’Ivoire, and the activity of various extracts were tested *in vitro* on various strains of *Plasmodium* (Koffi et al., 2020). The methanolic extract was the most active one. However, in Ghana, it was the decoction of *P. laxiflora* leave that was used against malaria (Asase et al., 2005). Although a putative antiviral activity may be due to different compounds and in different organs, our antiviral assays were carried out with leave extracts only.

**FIGURE 1:**
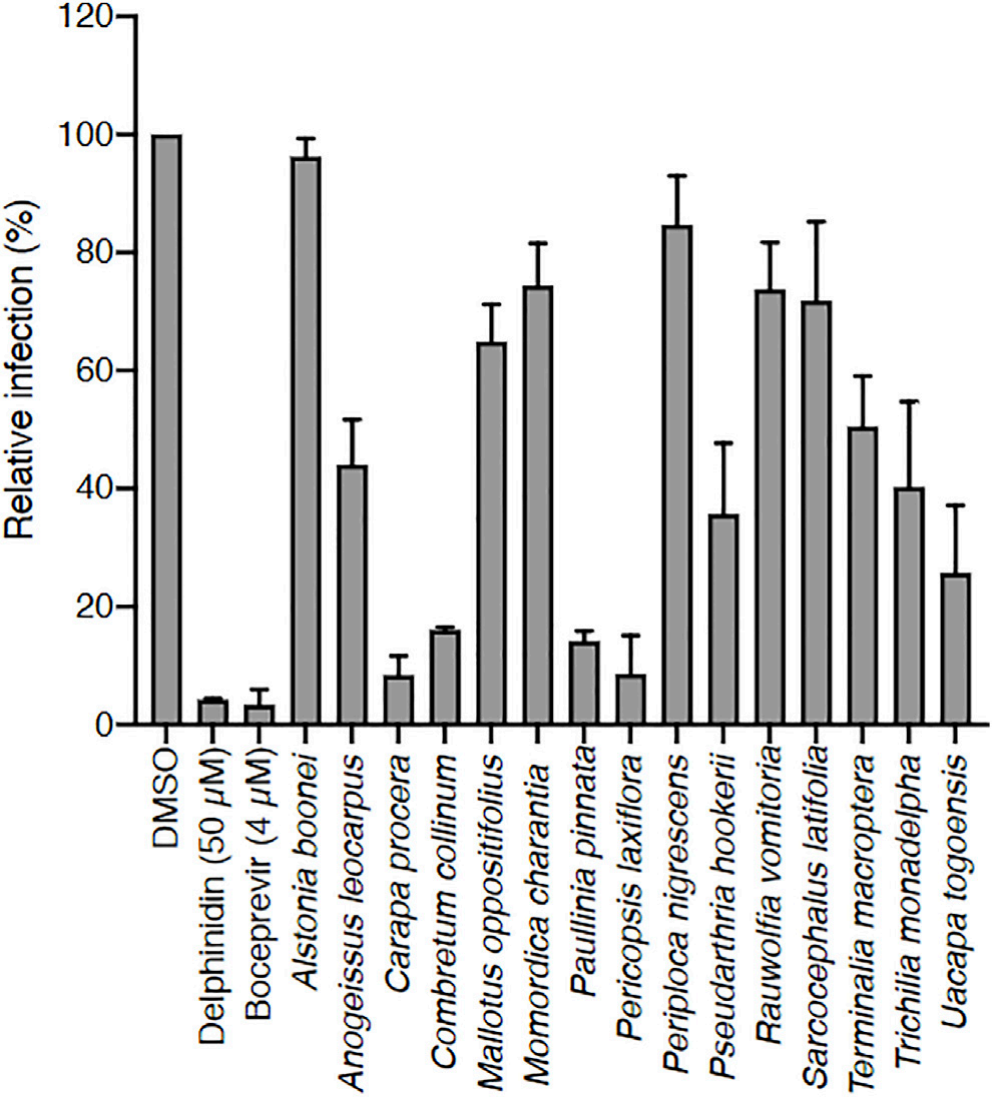
Screening of 15 Ivorian medicinal plant crude extracts for their anti-HCV activity. Each plant was extracted with methanol, and dried extracts were dissolved in DMSO (used here as negative control). Huh-7 cells were inoculated with HCV in the presence of crude extracts at 25 μg/ml, or delphinidin at 50 μM, or boceprevir at 4 µM. Infected cells were quantified 30 h post infection by immunofluorescence detection of viral E1 protein. Data are presented relative to untreated control. Data are means ± SEM of 3 experiments performed in triplicates.

Crude methanolic extracts of the 15 selected plants were prepared and tested *in vitro* on HCV infection (Figure 1). After removing methanol, the dried extracts were dissolved in DMSO at 25 mg/ml stock solution. Huh-7 cells were inoculated with HCV in the presence of the extracts at 25 μg/ml and infection was quantified using an immunofluorescent assay. DMSO was used as negative control, and delphinidin and boceprevir were used as positive controls. Indeed, delphinidin is a natural product with strong anti-HCV activity (Calland et al., 2015), and boceprevir is a commercial protease inhibitor used to treat hepatitis C. The cell number was also quantified at the end of the experiment to determine the toxicity of the extracts.

**FIGURE 2:**
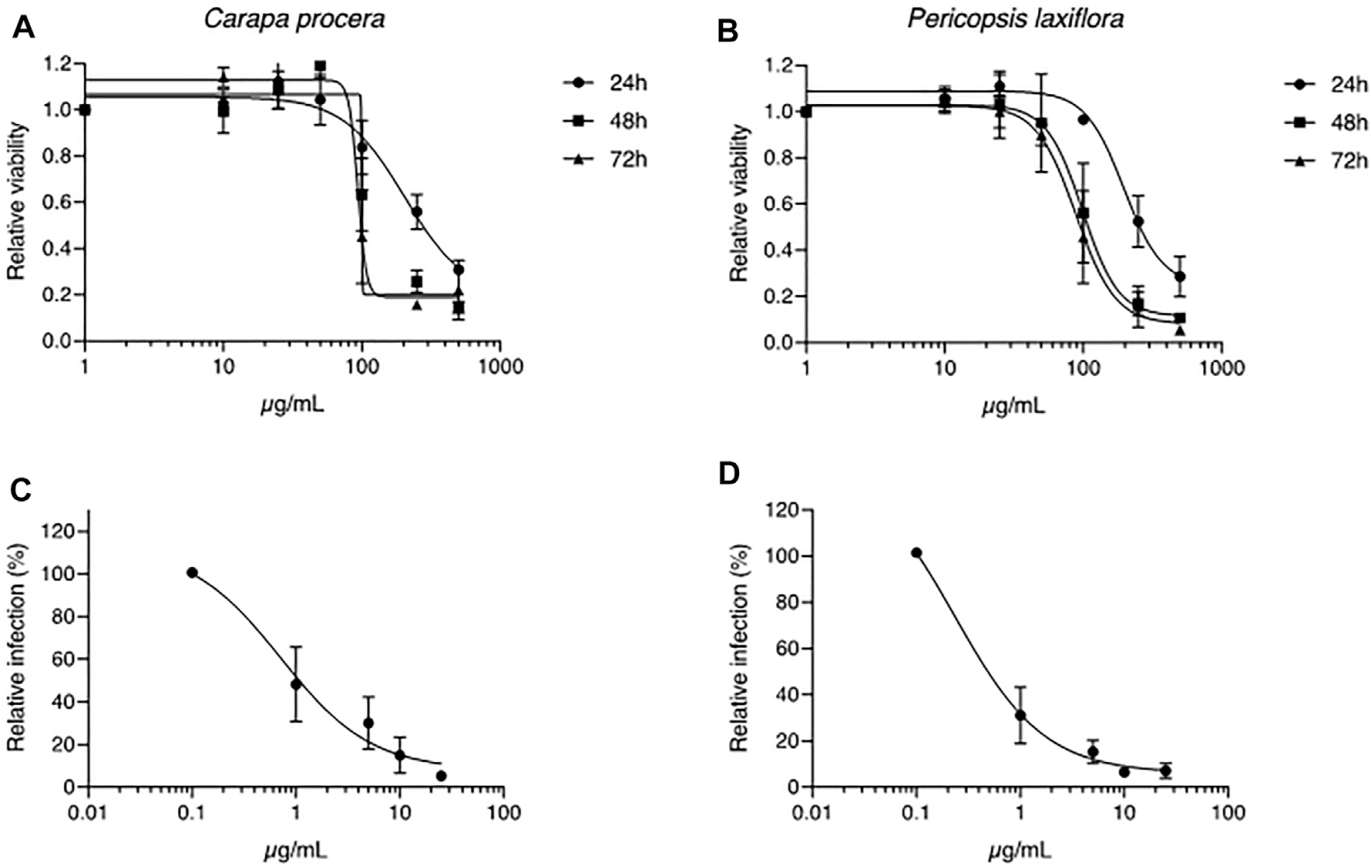
Toxicity and antiviral assays of *C. procera* and *P. laxiflora* crude extracts. **(A, B)**: Huh-7 cells were incubated with culture medium containing crude extracts at different concentrations. MTS toxicity assay was performed at 24, 48 or 72 h of incubation. **(C, D)**: Huh-7 cells were inoculated with HCV in the presence of crude extracts at different concentrations. Infected cells were quantified 30 h post infection by immunofluorescence detection of viral E1 protein. Data are presented relative to untreated control. Data are means ± SEM of 3 experiments performed in triplicates.

Among the 15 plants tested*, Carapa procera* DC. and *Pericopsis laxiflora* (Benth. ex Baker) Meeuwen extracts were generally the most active on HCV, with less than 10% infected cells when these were in contact with the corresponding crude extracts (25 μg/ml). In addition, these two extracts were not toxic on hepatic cells at those concentrations with a CC_50_ of 203.3 μg/ml and 196.6 μg/ml at 24 h, and 93.3 μg/ml and 88.7 μg/ml at 72 h for *C. procera* and *P. laxiflora*, respectively (Figures 2A,B). A dose-response concentration experiment was performed to determine the half-median inhibitory concentration IC_50_. A dose-response inhibitory activity was observed for the two extracts confirming their antiviral capacity against HCV. The IC_50_ was estimated at 0.71 and 0.23 μg/ml for *C. procera* and *P. laxiflora*, respectively (Figures 2C,D).

### Tannins Are Responsible for the Antiviral Activity of the Selected Plants

The chemical composition of *C. procera* and *P. laxiflora* extracts were evaluated by using TLC and conventional reagents to reveal major groups of secondary metabolites. Both extracts were rich in polyphenols, including flavonoids and tannins (Table 2). This is qualitatively consistent with previous results showing that leave extracts of *C. procera* were rich in polyphenols including anthocyanins and flavonols (Adjé et al., 2019). Polyphenol were also reported in *P. laxiflora* bark, and to a lesser extend in leave aqueous extracts (Alhaji et al., 2014). However, none of our extracts contained alkaloids, contrary to a previous study revealing some in *P. laxiflora* leave (Fadipe et al., 2019). This might be due to different extraction protocols.

**TABLE 2:** Phytochemical screening of the most active plants. Cp: *Carapa procera* DC.; Pl: *Pericopsis laxiflora* (Benth. ex Baker) Meeuwen; e: ethanol/water (50:50) eco-extract (crude methanolic extract otherwise); “−T”: after tannins removal; (−) absence or (+) presence of phytochemicals.

Metabolites \ extracts	Cp	Cp–T	eCp	eCp–T	Pl	Pl–T	ePl	ePl–T
Polyphenols	+++	+++	+++	+++	+++	+++	+++	+++
flavonoids	++	++	++	++	++	++	++	++
tannins	+	-	+	-	++	-	++	-
condensed tannins	+	-	+	-	++	-	++	-
anthraquinones	+	+	+	+	+	+	+	+
Terpenoids or steroids	+	+	+	+	+	+	+	+
Alkaloids	-	-	-	-	-	-	-	-

**FIGURE 3:**
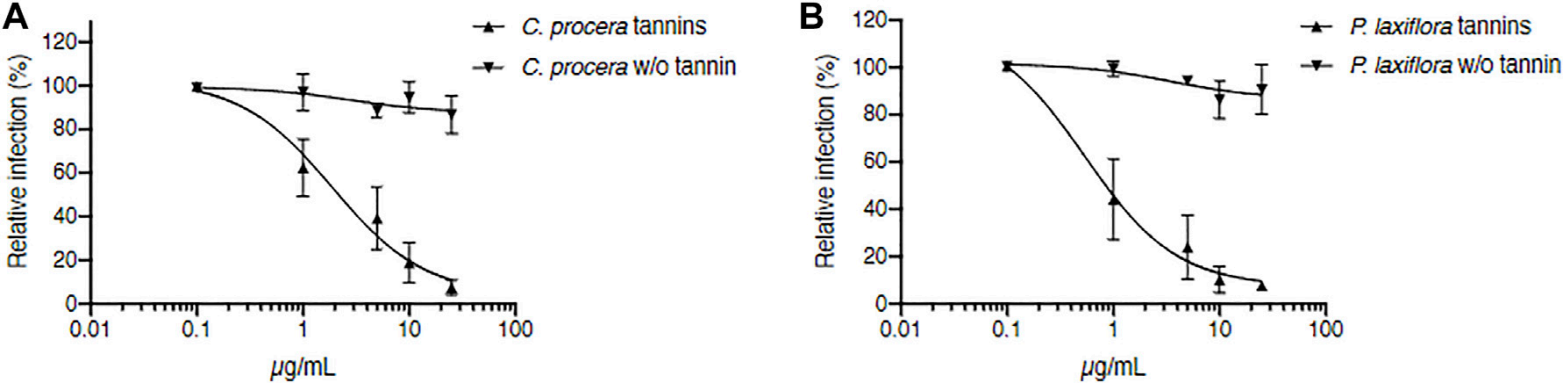
Tannins are responsible for *C. procera* and *P. laxiflora* activity against HCV. Tannin removal required heating of extracts. *C. procera* **(A)** and *P. laxiflora* **(B)** methanolic extracts were tested on HCV infection after heating (tannins), and after tannins removal (w/o tannins). Quantification of infection was as described above. Data are presented relative to untreated control and are means ± SEM of 3 experiments performed in triplicates.

Since many tannins have antiviral activity, especially against HCV (Zuo et al., 2007; Calland et al., 2012b; Lin et al., 2013; Ajala et al., 2014; Liu et al., 2015; Chowdhury et al., 2018; Tamura et al., 2019), they were then specifically removed in our two most active extracts (*C. procera* and *P. laxiflora)* in order to check whether these “tannin-free” extracts were still active against HCV or not. Results are presented in Figure 3.

The IC_50_ of crude extracts after heating were 1.89 and 0.53 μg/ml for *C. procera* and *P. laxiflora,* respectively. Although slightly higher, these were similar to the IC_50_ of the same extracts before heating (Figure 2C,D): 0.71 and 0.23 μg/ml for *C. procera* and *P. laxiflora*, respectively. Some active molecules might have been lost during this process, either by heat degradation (95°C), or because they were less soluble in water and therefore lost in the pellet (see material and methods section).

After heating and tannins removal (w/o tannin), both extracts lost completely their activity, regardless of their concentration (Figures 3A,B). Therefore, tannins are most likely responsible for *C. procera* and *P. laxiflora* activity against HCV *in vitro*.

### Traditional and Green Extractions of *C. procera* and *P. laxiflora* Preserve Their Antiviral Activity

**FIGURE 4:**
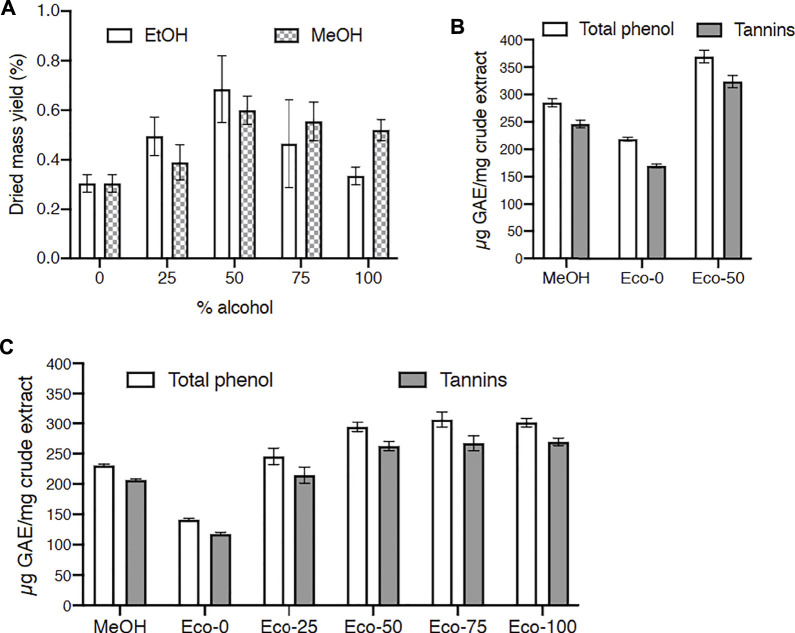
Effect of solvents on dried mass, total phenol and tannin yields for *Carapa procera* DC. and *Pericopsis laxifora* (Benth. ex Baker) Meeuwen. **(A)**: Dried mass yield for *C. procera* with hydro-alcoholic solvents containing 0–100% alcohol. **(B, C)**: Total phenol and tannin yields for *C. procera* **(B)** and *P. laxifora* **(C)**; GAE: gallic acid equivalent; Eco: hydroalcoholic eco-extracts with 0–100% ethanol; MeOH: crude methanolic extracts.

Our most active methanolic extracts were from *C. procera* and *P. laxiflora*. According to our ethnobotanical survey, *C. procera* and *P. laxiflora* leaves are traditionally used in water extracts. However, water may not be the best solvent to extract the active tannins of these plants. Acetone and alcoholic solvents as well as their mixtures with water have been commonly used to extract tannins from various vegetal sources (Wei et al., 2011; Metrouh-Amir et al., 2015). To avoid toxic solvents, water, ethanol and their mixtures were used here since they are considered as eco-friendly and traditionally used solvents. Hence, *C. procera* was extracted with hydro-alcoholic solvents containing 0–100% ethanol, and total polyphenols and tannins were quantified in these extracts. These results were compared to those obtained using similar water/methanol mixtures as solvents. The effect of these various hydro-alcoholic solvents was first studied on dried mass yields. Figure 4A shows that best yields were obtained with 50% alcohol (ethanol or methanol). The lowest yields were obtained with water or ethanol 100%. Moreover 50% ethanol extraction allowed a better yield than any (hydro-)methanolic extraction.

Then total polyphenols and tannins were quantified in these extracts (Figures 4B,C). For *C. procera* eco-extracts (Figure 4B), polyphenols and tannins contents increased when ethanol was added to water for extractions and reached a plateau from 50 to 100% ethanol. Quite similar solvent effect was reported for tannins extraction from *Arum maculatum* leaves (Farahmandfar et al., 2019). Since tannins are not very hydrophilic, the addition of ethanol in the solvent enhanced their extraction. Polyphenols and tannins contents were higher in these hydro-ethanolic extracts compared to methanolic extract. Similarly, for *P. laxiflora* (Figure 4C), the polyphenols and tannins contents were higher in the 50:50 hydro-ethanolic extract compared to methanolic and aqueous extracts.

**FIGURE 5:**
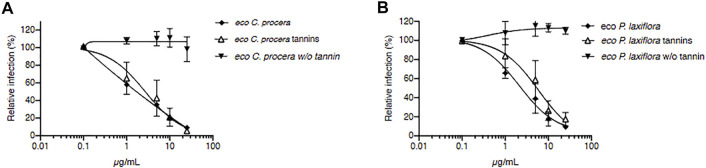
Tannin-rich eco-extracts of *C. procera* and *P. laxiflora* are active against HCV. *C. procera* **(A)** and *P. laxiflora* **(B)** eco-extracts (50% ethanol) were tested on HCV infection before tannin removal and before heating (squares), after heating (triangles), and after tannins removal (w/o tannins). Quantification of infection was as described above. Data are presented relative to untreated control. Data are means ± SEM of 3 experiments performed in triplicates.

Since the dried mass yields were better with the 50% hydro-alcoholic solvents compared to water or alcohol extractions (Figure 4), the ethanol/water (50:50) extracts were chosen for further antiviral assays. Figure 5 shows that for both *C. procera* and *P. laxiflora*, these crude hydro-ethanolic extracts were very active against HCV (IC_50_ = 1.18 and 2.11 μg/ml, respectively), even after heating (IC_50_ = 2.69 and 5.92 μg/ml, respectively). These results are similar compared to those of Figures 3A,B, suggesting that some active molecules might have been lost during the heating process before tannin removal. In addition, the ethanol/water (50:50) extract and crude methanolic extracts (Figure 3) had a similar anti-HCV activity, whereas the activity of the aqueous extracts was lower (Supplementary Figure S1). This is consistent with the tannin contents of all these extracts (Figures 4B,C). Moreover, the eco-extracts lost completely their activities after tannins removal, as the methanolic extracts did (Figure 3). Hence, the antiviral activities of *C. procera* and *P. laxiflora* are similar in eco-extracts compared to methanolic extracts, and they are also due to tannins. Those tannins are in much lower quantity in aqueous extracts that are less active against HCV. It is important to note that according to our survey, these plants were used in decoction, whereas our aqueous extracts were obtained at 20°C. Therefore, the compositions of these extracts were probably different. In this study our aim was to compare different solvents without heating.

The obtained results show that very active extracts could be obtained using eco-friendly solvents such as ethanol-water mixtures in a simple extraction and without heating or any further purification steps. Such extraction method respects the principles of the green extraction (Chemat et al., 2019) and could be easily applied for the preparation of antiviral extracts from *C. procera* and *P. laxiflora*.

### Procyanidin A2 and Other Tannins Contribute to the anti-HCV Activity of *C. procera* and *P. laxiflora* Extracts

Several extracts were analyzed by UPLC-MS especially for their tannins and anthocyanins that are known to have antiviral activities. For instance, epigallocatechin-3-gallate (EGCG) has been identified to inhibit HCV entry and cell-to-cell transmission (Ciesek et al., 2011; Calland et al., 2015). (−)-epicatechin-3-O-gallate (ECG) were found to inhibit NS3 protease in a study (Zuo et al., 2007), although it exhibited weak antiviral activity on HCV entry in another study (Calland et al., 2012a). Ellagic acid is also known to have anti-HCV activity (Reddy et al., 2014). Procyanidin B1, a dimer of (−)- epicatechin and (+)- catechin with a procyanidin type B structure, extracted from *Cinnamomi* cortex, could inhibit HCVpp entry in a dose-dependent manner (Li et al., 2010). More recently, a procyanidin with a type A structure was shown to inhibit HCV entry (Fauvelle et al., 2017). Results presented in Table 3 confirmed that all crude extracts contained tannins in various amounts, that were largely reduced—if not completely removed—after treating these samples with hide powder.

**TABLE 3:** Tannins and monomers content of *C. procera* and *P. laxiflora* extracts before and after tannin removal. For both plants, these molecules were searched in each extract (aqueous, hydro-ethanolic and methanolic) before (crude) and after treatment with hide powder (treated). +: presence; −: absence (no peak detected); (+): trace (peak area just above the noise). Bold: molecules found in all alcoholic (active) crude extracts but not in the treated ones.

Plants	*C. procera*						*P. laxiflora*					
molecules extracts	Aqueous		50% EtOH		MeOH		Aqueous		50% EtOH		MeOH	
	Crude	Treated	Crude	Treated	Crude	Treated	Crude	Treated	Crude	Treated	Crude	Treated
**procyanidin A1**	**(+)**	−	**(+)**	−	**(+)**	−	**(+)**	−	**+**	**(+)**	**+**	**(+)**
**procyanidin A2**	−	−	**(+)**	−	**(+)**	−	**+**	(**+)**	**++**	**+**	**++**	**+**
procyanidin B1	(+)	-	++	+	+	-	-	-	(+)	-	-	-
procyanidin B2	-	-	(+)	(+)	(+)	-	+	-	+	(+)	+	-
procyanidin B3	-	-	-	-	-	-	-	-	-	-	-	-
epicatechin	(+)	(+)	+	+	+	-	++	-	++	+	++	(+)
catechin	++	++	++	++	++	-	(+)	-	+	(+)	+	-
EGC	(+)	(+)	(+)	(+)	(+)	-	-	-	-	-	-	-
EGCG	-	-	-	-	-	-	-	-	-	-	-	-
**ellagic acid**	**(+)**	−	**+**	−	**(+)**	−	**(+)**	−	**+**	−	**+**	−

Some of these tannins or related molecules (precursors or degradation products) could contribute to the antiviral activity of these extracts. For instance, ellagic acid is present in all crude extracts, but absent after tannin removal. Conversely, other tannins (EGC and EGCG) or monomers (catechin and epicatechin) are either totally absent or present in all extracts of at least one plant, even after tannin removal. Thus, their contribution to the anti-HCV activity is unlikely here. More interestingly, at least traces of procyanidins A1 and A2 are present in most crude extracts, except procyanidin A2 which is absent in the *C. procera* aqueous extract (Table 3), but these compounds are not detected or in much smaller amounts after removing tannins with hide powder (“treated” extracts). Therefore, these tannins may explain the anti-HCV activity of *P. laxiflora* extracts at least, and to a lesser extend this of *C. procera* extracts, where those procyanidins are found in much smaller quantities. On the other hand, the activity of *C. procera* extracts might be due to tannins such as procyanidin B1, known for having effects on HCV replication (Li et al., 2010), and which is present in higher amounts for these crude extracts compared to those treated with hide powder. Similarly to the other dimers, procyanidin B2 was usually more concentrated in the most active (methanolic and hydro-ethanolic) extracts compared to the other ones (Table 3), and it has already shown antiviral activities on other viruses (Yang et al., 2014; Liu et al., 2018). Therefore, it might also contribute to the anti-HCV activity.

**FIGURE 6:**
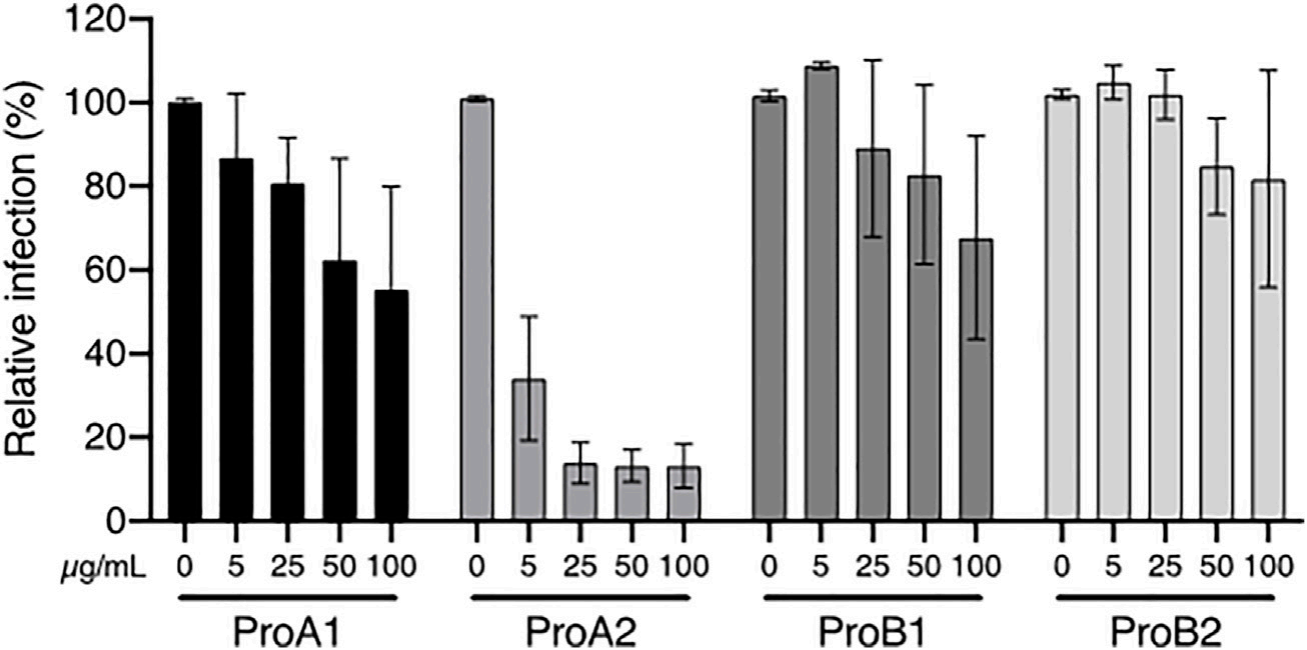
Anti-HCV activity of procyanidins. Huh-7 cells were inoculated with HCV in the presence of procyanidin A1 (ProA1), A2 (ProA2), B1 (ProB1), and B2 (ProB2) at 5, 25, 50 and 100 µM. Cells were fixed 30 h post-infection. Quantification of infection was as described above. Data are presented relative to untreated control and are means ± SEM of 3 experiments performed in triplicates.

Hence, these four procyanidins were tested against HCV. Their structures are shown in Supplementary Table S2. Figure 6 shows that procyanidin A2 is clearly the most active one, which is consistent with above results (Figures 3, 6; Table 3). For instance, extracts of *P. laxiflora* are antiviral before tannin removal, but not after. However, procyanidin A2 cannot explain the anti-HCV activity in all extracts. Indeed, this molecule is in much lower (trace) amount in *C. procera* extracts compared to *P. laxiflora* extracts, although the former plant has anti-HCV activity as well. Together with ellagic acid, procyanidins A1 and B1 may contribute to the activity of various extracts, although they are less active compared to procyanidin A2. Thus, the antiviral activity of all tested extracts is likely due to several tannins. Those that were found to be active were present in higher amounts in the most active extracts (methanolic and hydro-ethanolic), and also in aqueous extract in lower amount. In addition, they were absent from all these extracts after hide powder treatment. These results are correlated with the anti-HCV activity of these extracts: lower in aqueous extracts compared to alcoholic ones, and null after tannins removal.

Some of the tannins were identified and semi-quantified here, but many others were probably present in the same extracts. Indeed, the chromatograms in Supplementary Figure S2 shows that most peaks found in *C. procera* and *P. laxiflora* crude extracts can also be seen after tannin removal and therefore probably inactive on HCV. But, for both plants, the baseline is increased between 5- and 10-min elution for crude extracts, whereas it is close to zero after tannin removal. Thus, the difference is probably due to various tannins that were not all identified here and that may contribute to antiviral activity. Nonetheless, procyanidin A2 is one of the major compounds of *P. laxiflora* eco-extract, and was mostly removed after hide powder treatment (Supplementary Figure S2).

Tannins including procyanidins are generally known to have low oral biodisponibility. However, this depends on numerous factors, such as their degree of polymerization (DP). For example, procyanidins with DP < 4 can be absorbed and distributed in the organism, at least under certain conditions (Zhang et al., 2016). Depending on other factors (e.g. gastric pH, food intake), tannins can be degraded into smaller molecules, and metabolized by the colonic flora, leading to other compounds that may have a biological activity as well (Zhang et al., 2016; Smeriglio et al., 2017). In our survey, *P. laxiflora* was used in bath, whereas *C. procera* was also used in tea (Table 1). Therefore, digestion related factors might lead to other active molecules after drinking this plant extract. It would be interesting to test procyanidins and other putative anti-HCV tannins *in vivo*, before extrapolating any of our data in terms of clinical relevance for treating hepatitis C.

## Conclusion

Plants are widely used in Ivorian traditional medicine to treat various diseases. Our ethnobotanical survey revealed that at least 15 plants are used in the Bafing region to treat yellow malaria. Since this nosological category includes illnesses characterized by jaundice, some of these plants may have antiviral activity. Therefore their crude methanolic extracts were screened against HCV *in vitro*. The two most active ones, *Carapa procera* DC. and *Pericopsis laxiflora* (Benth. ex Baker) Meeuwen, were further studied for their activity (IC_50_: 0.71 and 0.23 μg/ml respectively), toxicity and chemical composition. They were not toxic on hepatic cells and rich in polyphenols including tannins. The latter were removed from these extracts before retesting on HCV, showing that tannins were responsible for antiviral activity. These active tannins could be efficiently extracted with eco-friendly and traditionally used hydro-ethanolic solvents, resulting in similar antiviral activities compared to methanolic extracts, and higher antiviral activity compared to aqueous extracts. Among them, procyanidin A2 was identified in several extracts and the pure molecule was very active in our *in vitro* antiviral assay. Other tannins most likely contributed to this activity. Further studies should be carried out to discover other active tannins and to investigate extracts activity *in vivo*.

## Data Availability

The raw data supporting the conclusions of this article will be made available by the authors, without undue reservation.

## Abbreviations

CC_50_, Half-median Cytotoxic Concentration; DAAs: Direct-Acting Antivirals; DAPI, 4′,6-diamidino-2-phenylindole; DMACA, *p*-dimethylaminocinnamaldehyde DMEM, Dulbecco’s modified Eagle’s medium; DMSO, Dimethyl sulfoxide; DP, Degree of Polymerization; ECG, (-)-epicatechin-3-O-gallate; EGCG, epigallocatechin-3-gallate; ELISA, Enzyme-Linked Immunosorbent Assay; EtOAc, Ethyl acetate; ffu, focus forming unit; HCV, Hepatitis C virus; HCVcc, HCV cell culture; Huh, Human hepatocyte; IC_50_: Half-median Inhibitory Concentration; IgG, Immunoglobulin G; JFH1, Japanese Fulminant Hepatitis-1; MOI, Multiplicity of infection; MTS, 3-(4,5-dimethylthiazol-2-yl)-5-(3-carboxymethoxyphenyl)-2-(4-sulfophenyl)-2H-tetrazolium; NS (protease/polymerase): Non Structural; PBS, Phosphate buffered saline; TLC, Thin Layer Chromatography; TPS, Total Phenolic Solution; TPS-T, TPS without tannins; UPLC, Ultra-High Performance Liquid Chromatography; w/o, without.
