# Supplementary figures and images for: Anti-HCV Tannins From Plants Traditionally Used in West Africa and Extracted With Green Solvents
Source: Front Pharmacol. 2022 Jan 28;12:789688. doi: 10.3389/fphar.2021.789688 (PMC8831738; doi:10.3389/fphar.2021.789688)

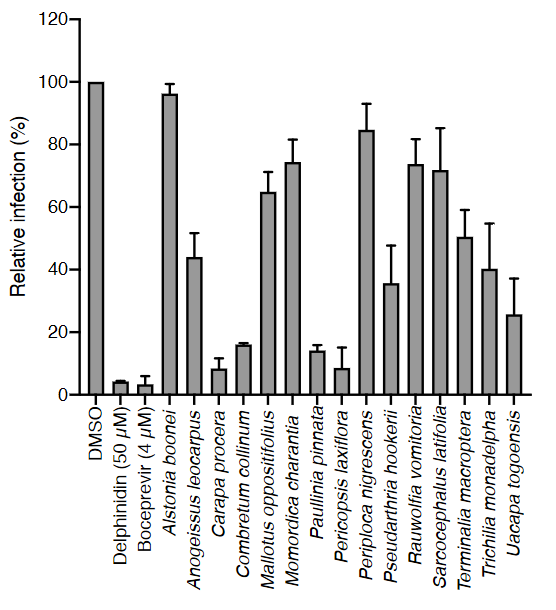

Supplement: Supplementary file 1 [file DataSheet1.ZIP › figures/Figure 1.tif]

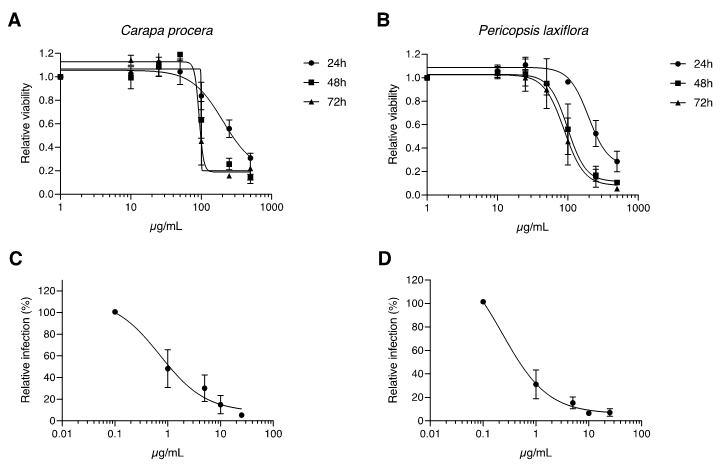

Supplement: Supplementary file 1 [file DataSheet1.ZIP › figures/Figure 2.tif]

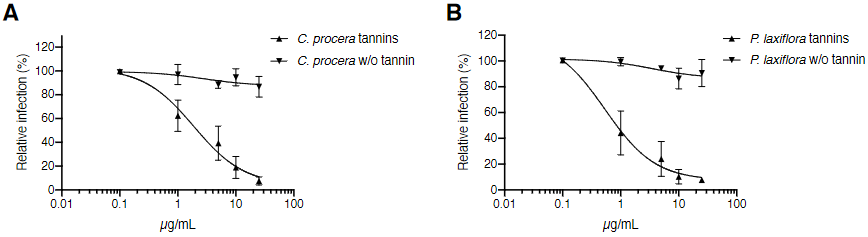

Supplement: Supplementary file 1 [file DataSheet1.ZIP › figures/Figure 3.tif]

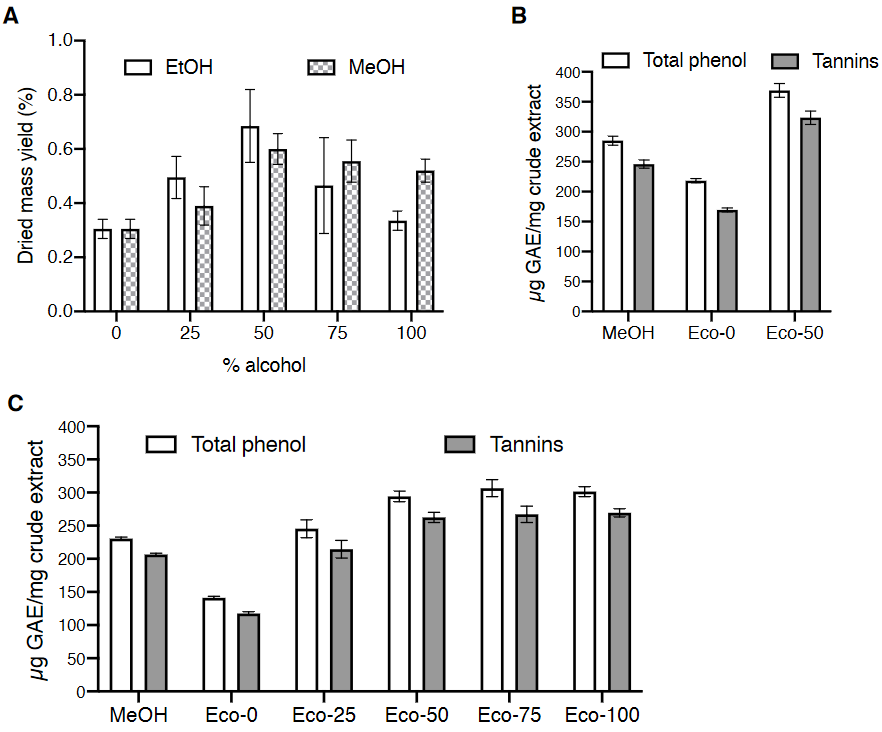

Supplement: Supplementary file 1 [file DataSheet1.ZIP › figures/Figure 4.tif]

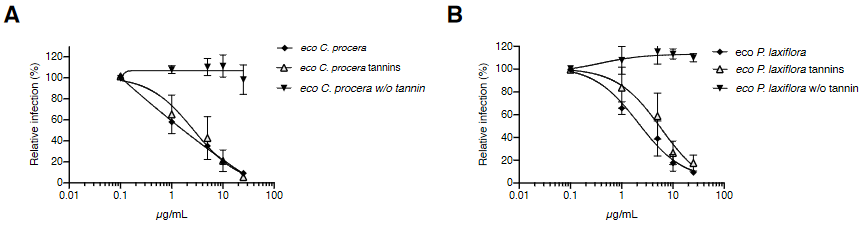

Supplement: Supplementary file 1 [file DataSheet1.ZIP › figures/Figure 5.tif]

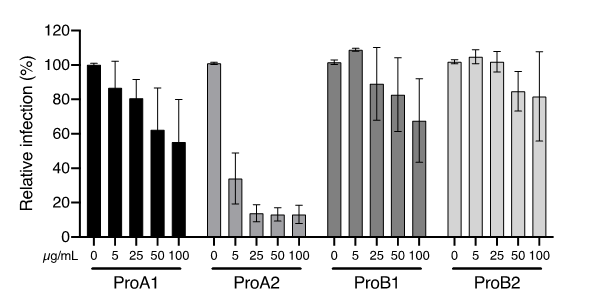

Supplement: Supplementary file 1 [file DataSheet1.ZIP › figures/Figure 6.tif]
