# Supplementary material for: Anti-HCV Tannins From Plants Traditionally Used in West Africa and Extracted With Green Solvents
Source: Front Pharmacol. 2022 Jan 28;12:789688. doi: 10.3389/fphar.2021.789688 (PMC8831738; doi:10.3389/fphar.2021.789688)
Supplement: Supplementary file 2 [file DataSheet2.docx]

Supplementary Material

# Supplementary Figures


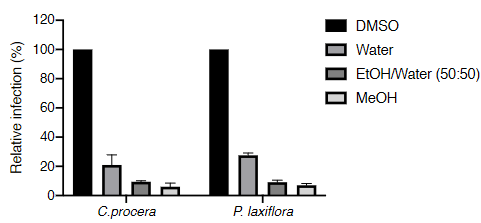


**Supplementary Figure 1:** Comparison of aqueous, hydro-ethanolic and methanolic crude extracts for their anti-HCV activity. Each plant was extracted with water, ethanol/water (50:50) or methanol, and dried extracts were dissolved in DMSO (used here as negative control). Huh-7 cells were inoculated with HCV in the presence of crude extracts at 25 µg/mL. Infected cells were quantified 30h post infection by immunofluorescence detection of viral E1 protein. Data are presented relative to untreated control. Data are means ± SEM of 3 experiments performed in triplicates.

**Supplementary Figure 2:** Representative chromatograms (280 nm) of *C. procera* (top) and *P. laxiflora* (bottom) eco-extracts (ethanol/water, 50:50), before (black) and after treatment with hide powder (blue). Procyanidin A2 is shown in red.

**Supplementary Table 1: UPLC data for the tannins and monomers semi-quantified in *C. procera* and *P. laxiflora* extracts**

|  | Tr (min) | m/z (-) | m/z (+) | λ Max (nm) |
| --- | --- | --- | --- | --- |
| procyanidin A1 | 4,074 | 575,38 | 577,17 | 211,8 & 279,3 |
| procyanidin A2 | 4,301 | 575,39 | 577,17 | 230,7 & 278,1 |
| procyanidin B1 | 3,333 | 577,39 | 579,21 | 207,1 & 279,3 |
| procyanidin B2 | 3,683 | 577,38 | 579,14 | 190,6 & 279,3 |
| procyanidin B3 | 3,369 | 577,40 | 579,17 | 207,1 & 279,3 |
| (-)-epicatechin | 5.809 | 289,18 | 291,05 | 234,2 & 278,1 |
| (+)-catechin | 4,635 | 289,18 | 291,07 | 231,8 & 279,3 |
| (-)-EGC | 4,152 | 305,18 | 307,04 | 230,7 & 269,8 |
| (-)-EGCG | 6,198 | 457,27 | 459,12 | 231,8 & 273,4 |
| ellagic acid | 7,193 | 301,11 | 302,98 | 198,8 ; 253,2 & 368,2 |

**Supplementary Table 2: Structures of the 4 procyanidins tested on HCV (from https://pubchem.ncbi.nlm.nih.gov/)**

| 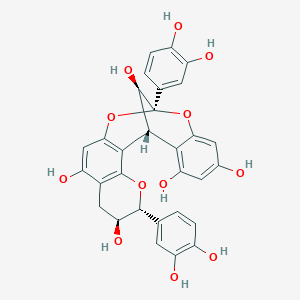  A1 | 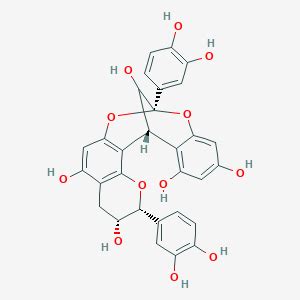  A2 |
| --- | --- |
| 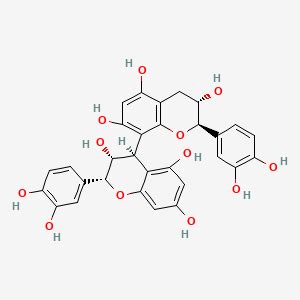  B1 | 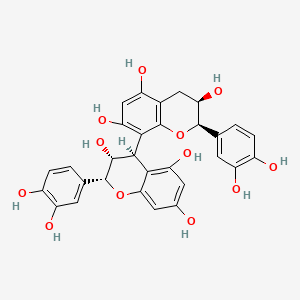  B2 |
